# Early recovery of cognition and brain plasticity after surgery in children with low-grade frontal lobe tumors

**DOI:** 10.3389/fped.2023.1127098

**Published:** 2023-03-10

**Authors:** Wenjian Zheng, Xueyi Guan, Xianchang Zhang, Jian Gong

**Affiliations:** ^1^ Department of Pediatric Neurosurgery, Beijing Neurosurgical Institute and Beijing Tiantan Hospital, Capital Medical University, Beijing, China; ^2^ Institute of Artificial Intelligence, Hefei Comprehensive National Science Center, Beijing, China; ^3^ MR Collaboration, Siemens Healthineers Ltd., Beijing, China

**Keywords:** Brain tumor, Cognition, Brain plasticity, rsfMRI, DTI

## Abstract

**Background:**

Low-grade frontal lobe tumors (LGFLT) can be cured through total resection, but surgical trauma could impair higher-order cognitive function. We aim to characterize the short-term natural cognitive recovery and brain plasticity in surgically-treated pediatric patients with LGFLT.

**Methods:**

Ten pediatric patients with LGFLT were enrolled. Their cognitive function was assessed before the surgery (S0), in the first month post-surgery (S1), and 3–6 months post-surgery (S2), using the CNS Vital Signs battery. DTI and rs-fMRI were performed during the same time periods. Changes of cognition and image metrics between S1>S0 and S2>S1 were analyzed.

**Results:**

The Motor Speed (MotSp) and Reaction Time (RT) scores significantly decreased in S1 and recovered in S2. Rs-fMRI showed decreased functional connectivity (FC) between the bilateral frontal lobes and bilateral caudates, putamina, and pallidi in S1>S0 (voxel threshold p-unc<0.001, cluster threshold p-FDR<0.05). In S2>S1, FC recovery was observed in the neighboring frontal cortex areas (p-unc<0.001, p-FDR<0.05). Among them, the FC in the caudates-right inferior frontal gyri was positively correlated to the RT (p-FDR<0.05). A DTI Tract-based spatial statistics (TBSS) analysis showed decreased fractional anisotropy and axial diffusivity mainly in the corticospinal tracts, cingulum, internal capsule, and external capsule at 0–6 months post-surgery (TFCE-p<0.05). The DTI metrics were not associated with the cognitive data.

**Conclusion:**

Processing speed impairment after an LGFLT resection can recover naturally within 3–6 months in school-age children. Rs-fMRI is more sensitive to short-term brain plasticity than DTI TBSS analysis. “Map expansion” plasticity in the frontal-basal ganglia circuit may contribute to the recovery.

## Introduction

In children, solid tumors occur most commonly in the brain. Moreover, 40–100% of pediatric patients with brain tumors suffer from a cognitive decline post-treatment (1). The frontal lobe is the second most frequent location for brain tumors in the pediatric population (16%) (2). Low-grade frontal lobe tumors (LGFLTs) could be cured by a total resection without additional adjuvant treatment. The development of the frontal lobe is relatively late. Its gray matter volumes peak at approximately 11–12 years of age, and the maturation is fully accomplished at the age of 25 years (3). Surgical insults to the frontal lobe could jeopardize higher-order cognitive functions, including executive function, decision-making, memory, emotional regulation, processing speed, and language (4). Thus, the benefit of an extended tumor resection should be balanced against the risk of cognitive impairments.

Several studies have confirmed that brain tumor surgery has a late effect on children’s cognitive function. Pletschko et al. reported that, after surgical treatment for cerebellar pilocytic astrocytoma, patients showed lower neurocognitive function than high academic achievers (mean follow-up period of 14 years) (5). Traunwieser et al. performed a cross-battery assessment at 2 and 5 years after the diagnosis of 316 pediatric patients with low-grade glioma and found that all survivors experienced long-term cognitive impairments in various domains (6). However, these long-term follow-up studies on brain tumor survivors had significant biases, such as the use of different treatment protocols and postoperative rehabilitation procedures. These biases may have been further amplified by differences in household income, parental education, and parental occupation (7).

A surgical injury of the frontal lobe in school-age children results in a significant brain plasticity, as the young brain undergoes rapid cell proliferation and axonal outgrowth (8). Hitherto, no study has reported the natural course of cognitive recovery and brain plasticity in surgically-treated pediatric patients with frontal lobe tumors. This study focused on short-term postoperative changes (0–6 months) in the cognition and brain networks of school-aged children (7–14 years) with LGFLT. We hypothesize that pediatric patients will show a rapid cognitive recovery and brain network plasticity after the frontal tumor surgery.

## Materials and methods

### Selection of the study participants

Ten pediatric patients at the Department of Pediatric Neurosurgery, Beijing Neurosurgical Institute and Beijing Tiantan Hospital were enrolled in the study from January 2020 to June 2021. The inclusion criteria for the study were: (1) Patients between 7 and 14 years old, (2) suspected of having low-grade brain tumors (localized tumors) according to preoperative MRI and CT, and (3) with no history of previous cerebral or systemic diseases. The exclusion criteria were: (1) Patients whose pathological results indicated a high-grade tumor (WHO classification III–IV) (9), and for whom (2) postoperative adjuvant treatments were demanded.

The surgery was performed by Dr. Jian Gong (Director of the Department of Pediatric Neurosurgery). Given the benign nature of the tumors, the aim of the surgery was gross total resection. Demographic information was recorded, and cognitive assessments (CNS Vital Signs, CNS VS), diffusion tensor imaging (DTI), and resting-state functional MRI (rs-fMRI) were conducted. All procedures were carried out in accordance with the relevant guidelines and regulations (the Declaration of Helsinki). The study was reviewed and approved by the Beijing Tiantan Hospital Institutional Review Board (KY2021-100-02). The patients’ guardians provided written informed consent for their participation in the study.

### Cognitive measures and analysis

Patients’ cognitive function was measured before the surgery (S0), in the first month after the surgery (S1), and 3–6 months after the surgery (S2). All tests were conducted by one of the authors (Xueyi Guan, a clinician in the Department of Pediatric Neurosurgery), who is responsible for conducting cognitive assessments of all patients in the department and has experience using CNS VS in over 100 pediatric patients. The CNS VS is a computer-administered neuropsychological assessment tool. It provides age-adjusted standard scores for 15 domains (10). These 15 domains include Composite Memory, Verbal Memory, Visual Memory, Psychomotor Speed, Complex Attention, Cognitive Flexibility, Executive Function, Social Acuity, Reasoning, Working Memory, Sustained Attention, Simple Attention, Reaction Time (RT), Processing Speed (PS), and Motor Speed (MotSp). Among them, MotSp was measured with the finger-tapping test. PS was assessed with the symbol digit coding test (SDCT). RT was measured with the Stroop test. The standard score for each domain had a mean of 100 and a standard deviation of 15. The test-retest correlation was reliable across the tested range of 3–156 days, which was suitable for a rapid perioperative assessment.

The data were analyzed with SPSS 17.0 (Chicago, IL, USA). Statistical comparisons of the means between the follow-up periods were carried out with paired Student’s t-test. A p-value<0.05 was considered significant. The Pearson product-moment correlation coefficient (r) was calculated as the strength of the linear association between domains and imaging metrics. An r value in the range 0.7–1.0, 0.5–0.7, 0.3–0.5, or <0.3 in the correlation analysis was regarded as “high correlation,” “moderate correlation,” “low correlation,” or “negligible/no correlation,” respectively (11). A p-value<0.05 was considered statistically significant. The results were displayed using “ggplot” in R.

### DTI acquisition and analysis

MRI was acquired on a 3T scanner (MAGNETOM Prisma, Siemens Healthcare, Erlangen, Germany) with a 20-channel head/neck coil. DTI was acquired using a single-shot spin-echo echo-planar image (SE-EPI) sequence. Diffusion gradients were applied along 64 directions using b-values of 0 and 3000 s/mm${}^{2}$. DWI data were obtained using the following parameters: (TR=2500 ms, TE=70 ms, $\text{flipangle}=90^{\circ }$, slices=68, % field of view (FOV)=90.6%, voxel size=2 mm isotropic, and no intersection gap). The imaging protocol included T1 weighted structure imaging with a magnetization prepared rapid acquisition gradient echo (MPRAGE) sequence (TR=1560 ms, TE=1.65 ms, $\text{flipangle}=8^{\circ }$, slices=176, % FOV=100%, voxel size=1 mm isotropic, and no intersection gap). The sections were approximately parallel to the anterior commissure-posterior commissure line.

The DTI data were analyzed using the standard procedure of the PANDA software (version 1.3.0) developed by Cui et al. (12), running in MATLAB R2016b version 9.1.0 (MathWorks, Inc., Natick, MA, USA). The processing procedures included skull stripping, a correction of the eddy current distortions, and building diffusion tensor models. The individual fractional anisotropy (FA) images of the native space were first registered to the FA template (FMRIB58_FA template) in the Montreal Neurological Institute (MNI) space, and then the resultant warping transformations were applied to resample the diffusion metrics into the MNI space of 2×2×2 mm (13). The diffusion metrics, including FA, mean diffusivity (MD), axial diffusivity (AD), and radial diffusivity (RD), were extracted for 20 white matter (WM) tracts identified from the WM probtract atlas provided by Hua et al. (14). The 20 white matter tracts include splenium, body and genu of corpus callosum, anterior, superior and posterior corona radiata, cingulum cortex, external capsule, anterior limb, posterior limb and retrolenticular part of internal capsule, cerebral peduncle, posterior thalamic radiation, tapetum, and cerebellar white matter, uncinate fasciculus, superior longitudinal fasciculus, sagittal stratum, and fronto-occipital fasciculus, and cerebellar peduncle. The normalized images were smoothed using an isotropic Gaussian kernel (6-mm full width at the half maximum).

The Tract-based spatial statistics (TBSS) (15) of the DTI metrics was performed between S1 versus S0 and S2 versus S1 using a general linear model through FSL and PANDA. A 5,000 repetition permutation test was conducted. Significant clusters were corrected using the threshold-free cluster enhancement method (TFCE, p<0.05). The results were visualized using FSLeyes (16). Correlations between the regional average values of the WM tracts and the cognitive data were calculated.

### Rs-fMRI acquisition and analysis

Patients’ rs-fMRI was performed at the same timepoints. Resting-state sequences were acquired with an echo-planar imaging (EPI) sequence. The scan parameters for the EPI sequence with a simultaneous multiscale acceleration technique were TR=2000 ms, TE=35 ms, slices=69, SMS=3, % FOV=100%, voxel size=2.2 mm isotropic, volumes=240, and no intersection gap. The patients were instructed to remain seated with their eyes closed. No sedation was applied during the examination. The data were analyzed using the CONN toolbox 20b (17) running on MATLAB R2016b version 9.1.0 (MathWorks, Inc., Natick, MA, USA).

In the CONN toolbox, the “default preprocessing pipeline for volume-based analyses” was used for rs-fMRI preprocessing. First, a slice-timing correction was performed. The slices were acquired in an ascending, interleaved order. The head motion, global brain signal, white matter signal, and cerebrospinal fluid signal were regressed out from the time course of the rs-fMRI.A tumor mask for each structural image was drawn by a neurosurgen. The damaged portion of the brain is masked during calculation of normalization parameters. The images were then normalized to a MNI template, and the normalized images were resliced with a target resolution of 2 mm. The normalized fMRI images were then smoothed (an 8 mm, full-width, half-maximum Gaussian kernel). The motion outlier threshold of the artifact detection tool was set at the 95^th^ percentiles in a normative sample with a motion threshold of 0.9 mm. An ART-based identification was applied, and acquisitions with displacement above the threshold were removed (18). A brain masking process was applied for a voxel-level analysis. A band-pass filter from 0.01 to 0.1 Hz was used to eliminate the physiological high-frequency cardiac and respiratory noise low-frequency drifts. Quality assurance plots were visually inspected to ensure the images were properly co-registered and transformed into the MNI space. Images, in which the movement in any direction exceeded either 3 mm translation or $3^{\circ }$ rotation, were excluded from the study. Images of two patients (the S1 images of subjects 8 and 9) were excluded due to massive head movements.

Following the preprocessing steps of rs-fMRI, seed-based functional connectivity (FC) maps were generated for each participant. Regions of interests (ROIs) were defined using the default atlas in the CONN toolbox. A total of 164 ROIs were used as the seeds. Among them, 132 ROIs were atlases of cortical and subcortical areas from the library Harvard-Oxford atlas and the automated anatomical labeling (AAL) atlas, whereas 32 ROIs were atlases of networks (19). FC pairs were obtained with 164 ROIs. Because the tumor mass effect and supplanted surgical residual cavity after the surgery could result in flawed co-registrations, we chose ROIs in the basal ganglia (the average value of the bilateral caudates, putamina, and pallidi) as seed points for whole-brain FC analysis. We investigated the FC difference among the S0, S1, and S2 timepoints using an unpaired t-test on the Fisher z-transformed score FC. Cluster-level inference was based on the random field theory, and a correction for multiple comparisons across the brain was conducted by controlling the false discovery rate (FDR) at both the voxel (p-unc<0.001) and cluster (p-FDR<0.05) levels using CONN’s implementation of the Benjamini-Hochberg algorithm (20). Correlations between the z-transformed score of FC in the basal ganglia and frontal lobes (18 ROIs including bilateral caudate, bilateral putamen, bilateral pallidum, and bilateral frontal pole, bilateral insular cortex, bilateral superior frontal gyrus, bilateral middle frontal gyrus, and bilateral inferior frontal gyrus, pars triangularis, and bilateral inferior frontal gyrus, pars opercularis) and cognitive domains in the CNS VS were calculated. FDR was applied in the correction for multiple comparisons.

## Results

Ten patients with low-grade frontal tumors and ages in the range of 7–14 years (mean age, 10.2±2.9 years) were included in the study. There were seven males and three females. The average tumor volume was 5.9±5.9 cm${}^{3}$. Patients’ information and clinical characteristics are presented in Table 1. The perioperative period was uneventful for all patients.

**Table 1:** Patients’ clinical characteristics.

No	Age/Gender	Pathology	Tumor location in MNI [x,y,z]	Volume (cm${}^{3}$)
1	13/Male	Glioma	Left corona radiata [−28,8,18]	1.1
2	6/Female	Dysembryoplastic neuroepithelial tumor	Right superior frontal cortex [22,18,50]	3.0
3	9/Male	Inflammatory granuloma	Left paracingulate cortex [−14,36,20]	3.8
4	13/Female	Epidermoid cyst	Right subcallosal cortex [5,16,−25]	3.1
5	13/Male	Ganglioglioma	Right superior frontal cortex [15,0,56]	0.8
6	8/Female	Cavernous hemangioma	Right middle frontal cortex [32,8,3]	16.8
7	14/Male	Glioma	Left middle frontal cortex [−22,28,30]	16.6
8	7/Male	Ganglioglioma	Right precentral cortex [34,−28,68]	2.7
9	11/Male	Cavernous hemangioma	Left precentral cortex [−36,−4,28]	4.9
10	8/Male	Dysembryoplastic neuroepithelial tumor	Left frontal pole cortex [−20,30,−12]	5.9

### Perioperative cognitive changes

**Figure 1:**
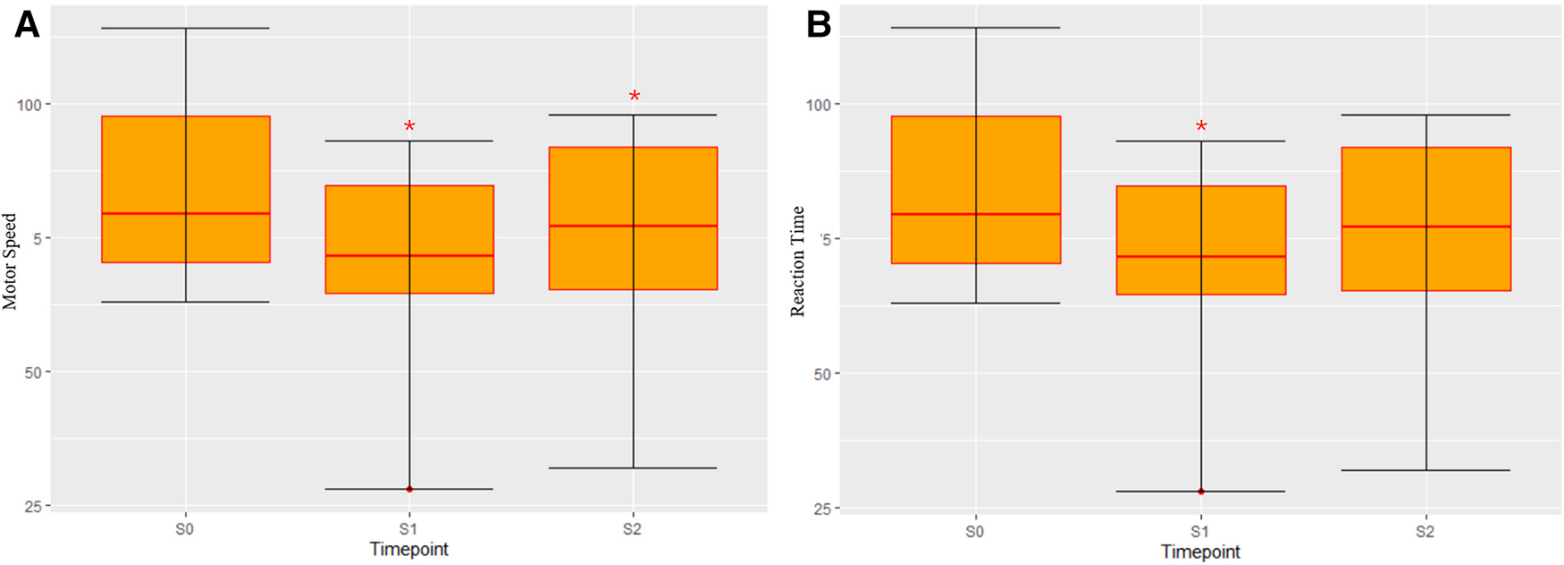
Changes in patients’ performance during the perioperative period. (**A**) Motor speed; (**B**) Reaction time. “*” indicates a significant statistical difference compared to the previous assessment (p<0.05). S0, before the surgery; S1, in the first month after the surgery; S2, 3–6 months after the surgery.

In the S0 baseline assessment, the domains most prominently decreased compared to the normative data were SocAcu (77.0±19.3, t=−3.55, p<0.01) and RT (84.3±16.7, t=−2.75, p=0.02). MotSp was within the normal range (96.6±19.3, t=−3.55, p=0.64). In the S1 assessment, MotSp (82.4±18.7, t=4.49, p<0.01) and RT (70.3±19.4, t=2.58, p=0.03) were significantly decreased. In the S2 assessment, MotSp (91.2±18.3, t=2.33, p=0.05) was significantly recovered, while RT (75.2±20.6, t=2.33, p=0.495) was slightly improved. The changes in MotSp and RT are elucidated in Figure 1.

### Perioperative white matter tract changes in DTI

**Figure 2:**
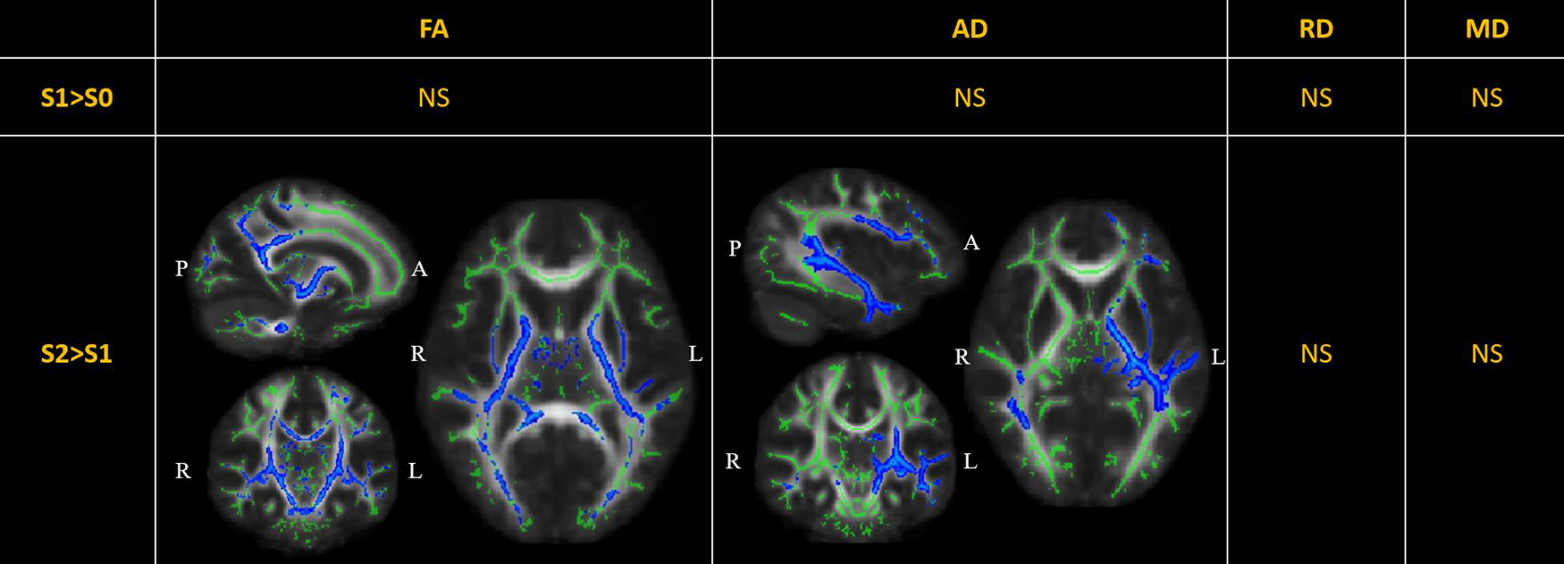
Perioperative changes in diffusion tensor imaging (DTI) metrics across S0, S1, and S2. In S2> S1, the FA of white matter (WM) tracts, including the bilateral corticospinal tracts (MNI [−15 −11 −5], peak t value=4.53), bilateral cingula (cingulate gyri) (MNI [15 −8 35], peak t value=3.22), bilateral internal capsules (MNI [−16 −10 1], peak t value=4.25), bilateral external capsules (MNI [−29 10 −1], peak t value=2.58), and left superior longitudinal fasciculus (MNI [−40 −17 28], peak t value=4.08), was significantly reduced; the AD was significantly reduced in the left superior-frontal blade (MNI [−42 −7 −14], peak t value=3.40), left parieto-temporal blade (MNI [−42 −28 -5], peak t value=4.48), left internal capsule (MNI [−21 −17 −3], peak t value=4.52), left external capsule (MNI [−31 7 −3], peak t value=2.91), and left corticospinal tract (MNI [−18 −17 −5], peak t value=3.27). The significant results (TFCE-p<0.05) are displayed as a blue color tract, using the “tbss_fill” script of FSL. The green tract represents the WM tract mask image used during randomized statistics. S0, before the surgery; S1, in the first month after the surgery; S2, 3–6 months after the surgery. FA, fractional anisotropy; AD, axial diffusivity; RD, radial diffusivity; MD, mean diffusivity; NS not significant.

DTI metrics comparisons are displayed in Figure 2. There were no significant changes in the diffusion metrics of the WM tracts between S0 and S1. Compared to S1, the FA was significantly reduced in S2 in nine out of 20 WM tracts, including the bilateral corticospinal tracts, bilateral cingula (cingulate gyri), bilateral internal capsules, bilateral external capsules, and left superior longitudinal fasciculus (TFCE-p<0.05). Further, the AD was significantly reduced in the left superior-frontal blade, left parieto-temporal blade, left internal capsule, left external capsule, and left corticospinal tract (TFCE-p<0.05). There were no significant changes in the RD and MD.

### Perioperative brain network changes in rs-fMRI

**Figure 3:**
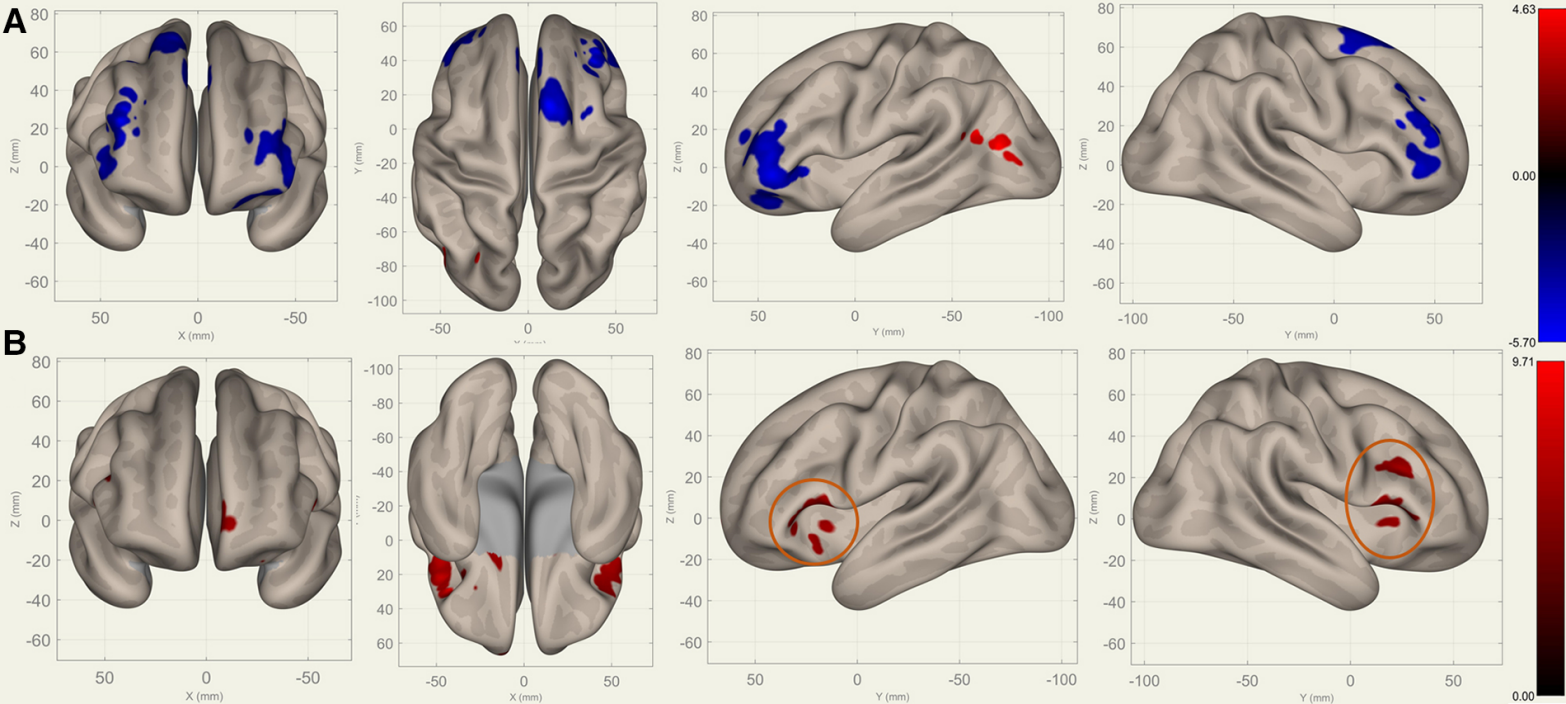
Perioperative seed-based connectivity changes in the bilateral caudates (seed points) (T>4.78, voxel threshold p-unc<0.001, cluster threshold p-FDR<0.05). (**A**) S1>S0: The decreases were most prominent in the left frontal pole (MNI [−38 +42 −10], cluster size: 605 voxels, peak p-unc<0.001, size p-FDR<0.001), right frontal pole (MNI [+38 +36 +28], cluster size: 441 voxels, peak p-unc<0.001, size p-FDR=0.001), right superior frontal gyrus (MNI [+10 +14 +62], cluster size: 338 voxels; peak p-unc<0.001, size p-FDR=0.002), and left superior frontal gyrus (MNI [−14 +30 +36], cluster size: 36 voxels, peak p-unc<0.001, size p-FDR=0.002). (**B**) S2>S1: The increases were most prominent in the left frontal orbital cortex (MNI [−20 +12 −12], cluster size: 260 voxels, peak p-unc<0.001, size p-FDR<0.001), right inferior frontal gyrus (MNI [+38 +22 +20], cluster size: 227 voxels, peak p-unc<0.001, size p-FDR=0.001), and left frontal operculum cortex (MNI [−44 +22 +12], cluster size: 182 voxels; peak p-unc<0.001, size p-FDR=0.002). The orange circles indicate “map expansion” neuroplasticity after the surgery.

**Figure 4:**
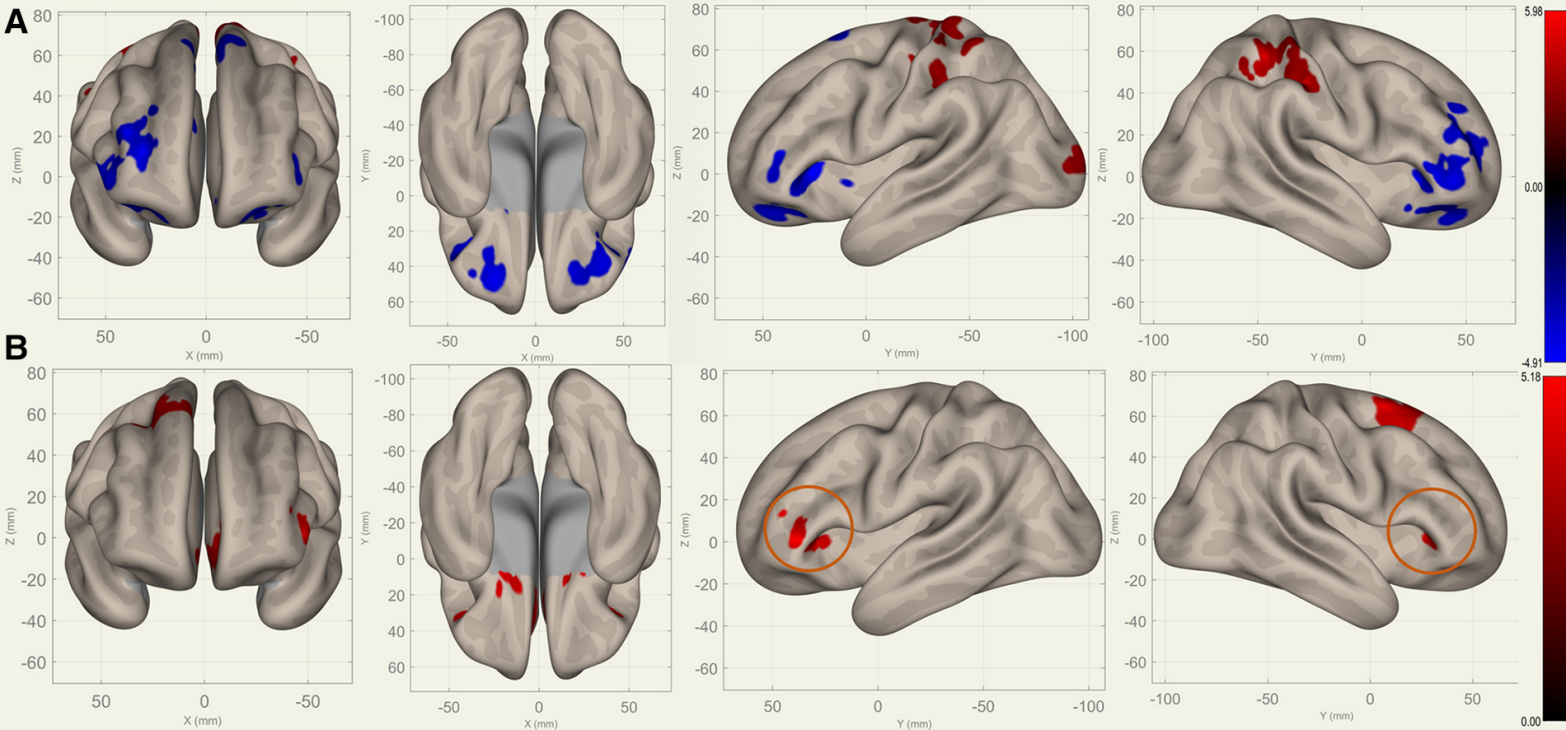
Perioperative seed-based connectivity changes in the bilateral putamina (seed points) (T>3.25, voxel threshold p-unc<0.01, cluster threshold p-FDR<0.05). (**A**) S1>S0: The decreases were most prominent in the right frontal pole (MNI [+40 +36 +00], cluster size: 1172 voxels, peak p-unc<0.001, size p-FDR<0.001), left frontal orbital gyrus (MNI [−30 +40 +06], cluster size: 852 voxels, peak p-unc<0.001, size p-FDR<0.001), and right paracingulate gyrus (MNI [+00 +40 +34], cluster size: 675 voxels, peak p-unc<0.001, size p-FDR<0.001). (**B**) S2>S1: The increases were most prominent in the left paracingulate gyrus (MNI [−18 +16 −08], cluster size: 1144 voxels; peak p-unc<0.001, size p-FDR<0.001) and the right superior frontal gyrus (MNI [+26 +10 +68], cluster size: 472 voxels; peak p-unc<0.001, size p-FDR<0.001). The orange circles indicate “map expansion” neuroplasticity after the surgery.

**Figure 5:**
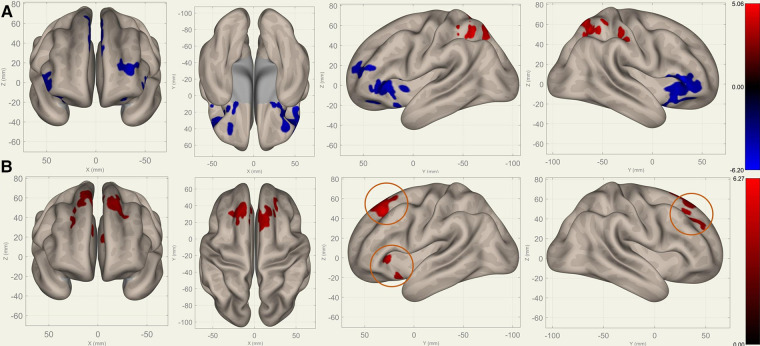
Perioperative seed-based connectivity changes in the bilateral pallidi (seed points) (T>3.25, voxel threshold p-unc<0.01, cluster threshold p-FDR<0.05). (**A**) S1>S0: The decreases were most prominent in the bilateral frontal poles (MNI [±10 +12 +00], cluster size: 2469 voxels, peak p-unc<0.001, size p-FDR<0.001) and the right superior frontal gyrus (MNI [+00 +24 +54], cluster size: 1289 voxels, peak p-unc<0.001, size p-FDR<0.001). (**B**) S2>S1: The increases were most prominent in the right superior frontal gyrus (MNI [+10 +36 +38], cluster size: 2004 voxels, peak p-unc<0.001, size p-FDR<0.001) and left frontal orbital cortex (MNI [−22 +22 +00], cluster size: 319 voxels, peak p-unc<0.001, size p-FDR=0.006). The orange circles indicate “map expansion” neuroplasticity after the surgery.

Compared to S0, S1 showed decreased FC in the bilateral frontal lobes between the bilateral caudates (Figure 3), putamina (Figure 4), and pallidi (Figure 5) (T>3.25, voxel threshold p-unc<0.01, cluster threshold p-FDR<0.05). Compared to S1, S2 demonstrated increased FC in the bilateral frontal lobes between the bilateral caudates (Figure 3), putamina (Figure 4), and pallidi (Figure 5). There were no significant differences between S0 and S2.

### Correlations between the cognitive data and imaging metrics

The analysis of the DTI metrics in 20 WM tracts (Table 2) revealed a significant positive correlation between the FA values in the right cingulum (hippocampus) and the RT scores (r=0.36, p-FDR<0.05). There was a significant positive correlation between the AD values in the left cingulum (cingulate gyrus) and the MotSp scores (r=0.38, p-FDR<0.05) as well as a significant positive correlation between the AD values in the right cingulum (hippocampus) and the RT scores (r=0.50, p-FDR<0.05).

**Table 2:** Correlations of the DTI metrics of 20 white matter tracts and cognitive results.

DTI metrics	FA		AD	
Cognitive results in the CNS VS	RT	MotSp	RT	MotSp
Anterior.thalamic.radiation.**L**	−0.08			
Anterior.thalamic.radiation.**R**	−0.06			
Corticospinal.tract.**L**				
Corticospinal.tract.**R**				
Cingulum.(cingulate.gyrus).**L**	−0.27		−0.13	0.38
Cingulum.(cingulate.gyrus).**R**	−0.09		−0.08	
Cingulum.(hippocampus).**L**				−0.29
Cingulum.(hippocampus).**R**	0.36	−0.19	0.5	−0.05
Forceps.major				−0.28
Forceps.minor	−0.07		−0.12	
Inferior.fronto-occipital.fasciculus.**L**	−0.13		−0.09	−0.04
Inferior.fronto-occipital.fasciculus.**R**		0.01		
Inferior.longitudinal.fasciculus.**L**				−0.08
Inferior.longitudinal.fasciculus.**R**		−0.19		
Superior.longitudinal.fasciculus.**L**			−0.14	
Superior.longitudinal.fasciculus.**R**	−0.14		−0.03	
Uncinate.fasciculus.**L**	−0.16			
Uncinate.fasciculus.**R**		−0.22		−0.11
Superior.longitudinal.fasciculus.(temporal.part).**L**		−0.19		−0.23
Superior.longitudinal.fasciculus.(temporal.part).**R**				

Spearman correlation coefficients (r) are shown in the matrix. Non-significant (p-FDR>0.05) coefficients are left blank. The red font indicates significant positive cognition-WH correlations. FA, fractional anisotropy; AD, axial diffusivity; RT, Reaction Time; MotSp Motor Speed.

The analysis of the FC z-transformed score between 18 ROIs of the basal ganglia-frontal lobes (Table 3) and 12 ROIs of the basal ganglia-motosensory cortex (Table 4) revealed that the RT was primarily correlated with the FC in the caudates, including the caudate-bilateral insular cortices (IC) (p-FDR<0.05), caudate-bilateral inferior frontal gyri (IFG) (p-FDR<0.05), and caudate-superior frontal gyri (SFG) (p-FDR<0.05). The RT was also positively correlated with the FC in the putamen-left middle frontal gyrus (MFG) (r=0.34, p-FDR<0.05) and putamen-right IFG (pars triangularis) (r=0.26, p-FDR<0.05).

**Table 3:** Correlations of the functional connectivity z-transformed score of basal ganglia-frontal lobes and cognitive data.

ROIs	Caudate				Putamen				Pallidum			
Lateral	Right		Left		Right		Left		Right		Left	
CNS VS	RT	MotSp	R	MotSp	RT	MotSp	RT	MotSp	RT	MotSp	RT	MotSp
FP r										−0.25	−0.25	
FP l						−0.15		−0.32	−0.11		−0.14	−0.26
IC r	0.24		0.23									
IC l	0.36		0.24									
SFG r			0.54							−0.37		
SFG l	0.33					−0.44		−0.25		−0.24	−0.18	
MidFG r				−0.14						−0.33		−0.24
MidFG l	0.38				0.34			−0.13				
IFG tri r	0.26	−0.3			0.26	−0.19				−0.41		
IFG tri l	0.35					−0.29		−0.3			−0.13	
IFG oper r	0.28		0.33									
IFG oper l	0.45											

Spearman correlation coefficients (r) are shown in the matrix. Non-significant (p-FDR>0.05) coefficients are left blank. The red font indicates significant positive correlations in both cognitive data and functional connectivity analysis. An r closer to ±1 indicates a stronger correlation. Abbreviations: FP r (Frontal Pole Right), FP l (Frontal Pole Left), IC r (Insular Cortex Right), IC l (Insular Cortex Left), SFG r (Superior Frontal Gyrus Right), SFG l (Superior Frontal Gyrus Left), MidFG r (Middle Frontal Gyrus Right), MidFG l (Middle Frontal Gyrus Left), IFG tri r (Inferior Frontal Gyrus, pars triangularis Right), IFG tri l (Inferior Frontal Gyrus, pars triangularis Left), IFG oper r (Inferior Frontal Gyrus, pars opercularis Right), IFG oper l (Inferior Frontal Gyrus, pars opercularis Left).

**Table 4:** Correlations of the functional connectivity z-transformed score of basal ganglia- motor sensory cortex and cognitive data.

ROIs	Caudate				Putamen				Pallidum			
Lateral	Right		Left		Right		Left		Right		Left	
CNS VS	RT	MotSp	RT	MotSp	RT	MotSp	RT	MotSp	RT	MotSp	RT	MotSp
PreCG r	0.09					−0.28		−0.2		−0.11		
PreCG l		−0.04	0.20		−0.21	−0.09			−0.08			
PostCG r	0.11					−0.35		−0.21				
PostCG l	0.16	−0.13	0.22			−0.23		−0.19	−0.03			
SMA r	0.23		0.24		−0.25	−0.26	−0.16		−0.05			
SMA l	0.06	−0.31	0.2		−0.4		−0.49		−0.23		−0.33	

Spearman correlation coefficients (r) are shown in the matrix. Non-significant (p-FDR>0.05) coefficients are left blank. An r closer to ±1 indicates a stronger correlation. Abbreviations: Abbreviation: PreCG r (Precentral Gyrus Right), PreCG l (Precentral Gyrus Left), PostCG r (Postcentral Gyrus Right), PostCG l (Postcentral Gyrus Left), SMA r (Supplementary Motor Cortex- Right), SMA L (Supplementary Motor Cortex- Left).

Compared to the RT, the MotSp showed a widespread negative association with the connectivity in the basal ganglia. In the FC of the basal ganglia-motosensory cortex (Table 4), MotSp was negatively correlated with the FC in the caudate-left SMA (r=−0.31, p-FDR<0.05) and putamen-right SMA (r=−0.26, p-FDR<0.05). In the basal ganglia-frontal lobe (Table 3), it was also negatively correlated with the FC in the basal ganglia-IFG (p-FDR<0.05), putamen-frontal pole (p-FDR<0.05), putamen-right SFG (p-FDR<0.05), and pallidum-right SFG (p-FDR<0.05).

## Discussion

We observed an early (baseline-postoperative to 3rd–6th month) cognitive recovery and functional plasticity in school-age children with LGFLT after total tumor resection surgery. This recovery reveals the natural course of their self-repair ability, without the interference of rehabilitation or adjuvant therapy. To detect such early changes, which could be minor, we used a computerized neurocognitive battery recording reaction time with millisecond precision. We also applied DTI and rs-fMRI to reveal structural and functional alternations, respectively. It has been established that both techniques can identify brain plasticity (21). They measure two different re-organization mechanisms, namely “neural plasticity” and “functional plasticity”. In children with LGFLT, we found a gap between the functional plasticity measured by rs-fMRI and microstructural plasticity in the WM tracts measured by DTI.

### Frontal tumor surgery impaired the information processing speed

The information processing speed is an important predictor of academic achievement (22). An assessment of the information processing speed should include motor and non-motor domains. In the CNS VS battery, MotSp represents a simple motor reaction, while PS and RT involve both motor and cognitive elements of the reaction (23). In S0, RT was significantly impaired. Previous research demonstrated that RT is sensitive to frontal lobe damage (24) and showed an association of structural MRI with the myelin integrity of the frontal lobes (25). A resting-state EEG study also indicated that prefrontal electrical activity predicted the RT (26). Our findings in pediatric patients with LGFLT were consistent with previous reports. However, we found that the MotSp remained at normative levels in S0. This may have been because the simple voluntary motor speed of the hands is related primarily to the SMA (27). The SMA is located on the dorsomedial aspect of the superior frontal gyrus, with the prefrontal sulcus and primary motor cortex as the posterior border. It is involved in the “Go and Stop” process of motor movement (28). Since in our study the tumors of only two patients affected the SMA, the MotSp was not significantly decreased in S0.

Surprisingly, the PS was not impaired at the baseline and remained stable after the surgery (S0=103.7±18.1 versus S1=100.0±9.7, p=0.470). The PS was measured using the SDCT, which is associated with the prefrontal cortex, hippocampus, and superior temporal gyrus (29). The results indicated that the SDCT might not have been sensitive to the insult from the frontal lobe tumor.

Because brain tumors can lead to functional retention within the tumor and functional reorganization in the adjacent cortex (30), an LGFLT resection can further damage the RT. This theory corresponds with the significant RT decrease, which was observed in S1>S0. Notably, brain surgery per se can cause edema in the peri-tumoral tissue and may affect larger cortex areas than the tumor invasion (31). In addition, biochemical (e.g., intracranial pressure and neurotransmitter release) and genomic alternations (e.g., protein synthesis) after brain surgery can lead to the inhibition of distant brain regions (32). In the rs-fMRI analysis, we observed decreased FC between the bilateral SMA (superior frontal gyrus) and bilateral caudate/putamen/pallidum (Figures 2–4). This can explain the corresponding MotSp decrease in S1.

### Functional neuroplasticity in reaction time and the frontal-basal ganglia circuit

We found a dramatic and rapid recovery of the MotSp in S2, which was approaching the baseline level. The RT also slightly recovered. This phenomenon has been reported in adult patients with brain tumors, in whom the immediate postoperative motor or language function would worsen transiently and recover within 1–3 months (33). The mechanism of the rapid recovery of processing speed is yet to be established. Below, we discuss the basal ganglia-frontal lobes connectivity changes during S0–S1–S2.

Basal ganglia interact closely with the frontal cortex. The frontal-basal ganglia circuit plays a critical role in attention and working memory (34). The basal ganglia surround the diencephalon and are made up of five subcortical nuclei: pallidum, caudate, putamen, substantia nigra, and the subthalamic nucleus of Luys. The caudate and putamen (i.e., the neo-striatum) are the input nuclei of the basal ganglia, which receive afferent input from the motor cortex of the frontal lobes through the cortico-striatal projections. The pallidi are the major output nuclei of the basal ganglia. They are involved with gating incoming sensory input to higher motor areas to coordinate behavioral responses (35). Parallel loops of the neostriatum connect the frontal lobe and the thalamus. Basal ganglia act as a “brake release” for motor actions on the frontal cortex (36).

Figures 3–5 illustrate the FC changes in the three pairs of core nuclei in the basal ganglia. In S1>S0, the FC decreased in the bilateral frontal lobes. This can be explained by trauma from the tumor resection and postoperative edema. In S2>S1, FC recovery was observed in the frontal lobes, where the recovery “shifted posteriorly” from the injured site—the cortex, which showed decreased FC in S1>S0. This phenomenon, consisting of an initially decreased FC in the peritumoral cortex areas and subsequently increased FC in the neighboring cortex areas, may represent the typical “map expansion” neuroplasticity described by Grafman (37). The term ”map expansion” describes that the pool of neurons that respond to behaviorally relevant stimuli expands, to accomplish the task more efficiently (38).

The associations between these “map expansion” cortex areas and cognitive data were verified in a correlation analysis. Among the ROI-ROI with significantly increased FC in S2>S1 (Figure 3 and Table 3), the FC in the caudates-right IFG was positively correlated to the RT (r=0.26-0.33) in the correlation analysis. We also found that the RT was positively correlated to the majority of ROI-ROI connectivity related to the caudate. This indicated that new neural circuits were emerging, as the original cortical areas had been destroyed by the tumor and surgical trauma. This finding provided evidence that “map expansion” neuroplasticity can happen as early as 3–6 months after frontal tumor surgery in children.

### Rapid restoration of the motor speed

We did not find any positive relationship between the MotSp and connectivity in the frontal lobes in the ROI-ROI analysis. As alluded to earlier, MotSp is associated with the SMA activities in healthy subjects. In our study, only two patients had tumors offending the SMA (subjects No. 2 and 5). We supposed that the SMA was mainly affected by preoperative tumor edema (in the S0) and transient postoperative edema (in the S1). We compared the MotSp change between the patients who had significant postoperative edema in the S1 (larger edema volume in the S1 than that of the S0) and those who had not. The result showed that patients without edema (subject No. 1,2,3,6,7,8 and 9) gained a larger improvement in S2 than those having edema (subject No. 4,5 and 10) (13.0±10.5 vs −1.0±10.4, Mann-Whitney test Z=−1.943, two-tailed p=0.052). This indicated that postoperative edema plays a negative effect on MotSp improvement. As the edema subsided, the MotSp rapidly restored to the preoperative level in the S2. The restoration may primarily rely on the recovery of the original neural circuit (basal ganglia-SMA), which had been interrupted but not destroyed.

In the correlation analysis, however, our results showed that the MotSp negatively correlated with the FC of the caudate-SMA (r=−0.31) and the putamen-SMA (r=−0.26) (Table 4). The underlying mechanism of this counterintuitive finding is less clear. One explanation is that the frontal lobe remapping rewrote the functional architecture of the SMA (39). The SMA became more “crowded,” and the activation in the basal ganglia-SMA circuit now represented new allocated functions (attention and processing speed) rather than the motor speed of the patients.

### White matter tract changes

DTI metrics reportedly reflect the integrity of WM tracts and have been applied as neuroimaging biomarkers in a range of cerebral diseases (40). Dennis et al. have investigated WM tract integrity in over 500 children and adolescents with traumatic brain injury (TBI) during acute/subacute (<2 months), post-acute (2–6 months), and chronic post-injury periods (≥6 months). They found significantly lower FA and higher MD in those with TBI compared to the controls during all three periods (41). In our DTI analysis, LGFLT resection led to extensively decreased FA and AD in WH tracts from 0 to 6 months after surgery. The major affected WM tracts were the corticospinal tracts, cingulum, internal capsule, and external capsule (Figure 2). Our results were similar with those of Dennis et al. (41), indicating that tumor resection is a special form of TBI.

FA has been interpreted as a proxy for myelin integrity. Most TBI studies have agreed that decreases in the FA and AD parallel shearing and disintegrated WM structures (42), while AD is less sensitive to pathologic changes from the acute to the chronic stage (43). In our study, the decreased FA and AD were not associated with the rebound of cognitive measurements in S2 (Figure 2 and Table 2). Although a correlation analysis showed that the RT was positively correlated to the FA and AD values in the right hippocampus cingulum (r=0.36-0.50) (Table 2), the corresponding WM tract did not show significant changes in either S1>S0 or S2>S1 (Figure 2). It has been previously demonstrated that the anterior cingulate cortex is responsible for executive functions and the processing speed, whereas the hippocampus cingulum is related to memory (44). However, in pediatric patients with LGFLT, the RT was significantly associated with the hippocampus cingulum rather than with the cingulate cortex. It is plausible that preoperative reorganization of the WM tracts occurred due to tumor disruption or compression in the frontal lobes. While interrupted by a cerebral injury (e.g. tumor invasion or brain surgery), the neuroanatomical changes may not necessarily translate to functional presentations in young brains (45). The above results suggest that DTI is not sensitive enough to predict cognitive changes after surgery in pediatric patients with LGFLT.

### Limitations

The major limitation of our study was that the sample size was relatively small. The tumor size and affected cerebral cortex areas varied, which precluded the direct assessment of rs-fMRI or DTI metrics in the specific cortex areas of the frontal lobes.

## Conclusions

This is a longitudinal study on short-term (0–6 months) postoperative changes in cognition, DTI parameters, and rs-fMRI parameters in surgically-treated pediatric patients with LGFLT. The resection of frontal tumors impaired the processing speed, and the impairment recovered without an intervention within 3–6 months after the surgery. While TBSS analysis in DTI was not sensitive to short-term brain plasticity in patients with LGFLT,functional connectivity analysis in rs-fMRI showed that “map expansion” phenomenon in the frontal-basal ganglia circuit. This plasticity might have contributed to the cognitive recovery. The frontal-basal ganglia circuit could be a potential therapeutic target for pediatric patients with LGFLT during early postoperative rehabilitation.

## Data Availability

The raw data supporting the conclusions of this article will be made available by the authors, without undue reservation.

## References

[1] Duffner PK. Risk factors for cognitive decline in children treated for brain tumors. Eur J Paediatr Neurol. (2010) 14:106–15. 10.1016/j.ejpn.2009.10.005 19931477

[2] Zumel-Marne A Kundi M Castano-Vinyals G Alguacil J Petridou ET Georgakis MK, et al. Clinical presentation of young people (10–24 years old) with brain tumors: results from the international mobi-kids study. J Neurooncol. (2020) 147:427–40. 10.1007/s11060-020-03437-4 32124185 PMC7136306

[3] Arain M Haque M Johal L Mathur P Nel W Rais A, et al. Maturation of the adolescent brain. Neuropsychiatr Dis Treat. (2013) 9:449–61. 10.2147/ndt.S39776 23579318 PMC3621648

[4] Fang S Wang Y Jiang T. The influence of frontal lobe tumors, surgical treatment on advanced cognitive functions. World Neurosurg. (2016) 91:340–6. 10.1016/j.wneu.2016.04.006 27072331

[5] Pletschko T Felnhofer A Lamplmair D Dorfer C Czech T Chocholous M, et al. Cerebellar pilocytic astrocytoma in childhood: investigating the long-term impact of surgery on cognitive performance, functional outcome. Dev Neurorehabil. (2018) 21:415–22. 10.1080/17518423.2017.1370502 28968151 PMC6050644

[6] Traunwieser T Kandels D Pauls F Pietsch T Warmuth-Metz M Bison B, et al. Long-term cognitive deficits in pediatric low-grade glioma (LGG) survivors reflect pretreatment conditions-report from the German LGG studies. Neurooncol Adv. (2020) 2:vdaa094. 10.1093/noajnl/vdaa094 32968720 PMC7497816

[7] Jirout J LoCasale-Crouch J Turnbull K Gu Y Cubides M Garzione S, et al. How lifestyle factors affect cognitive and executive function and the ability to learn in children. Nutrients. (2019) 11(8):1953. 10.3390/nu11081953 31434251 PMC6723730

[8] de Ruiter MA van Mourik R Schouten-van Meeteren AY Grootenhuis MA Oosterlaan J. Neurocognitive consequences of a paediatric brain tumour, its treatment: a meta-analysis. Dev Med Child Neurol. (2013) 55:408–17. 10.1111/dmcn.12020 23157447

[9] Louis DN Perry A Reifenberger G von Deimling A Figarella-Branger D Cavenee WK The 2016 World Health Organization classification of tumors of the central nervous system: a summary. Acta Neuropathologica. (2016) 131(6):803–20. 10.1007/s00401-016-1545-1 27157931

[10] Gualtieri CT Johnson LG. Reliability, validity of a computerized neurocognitive test battery, CNS Vital Signs. Arch Clin Neuropsychol. (2006) 21:623–43. 10.1016/j.acn.2006.05.007 17014981

[11] Mukaka MM. Statistics corner: a guide to appropriate use of correlation coefficient in medical research. Malawi Med J. (2012) 24:69–71. 10.4314/MMJ.V24I3 23638278 PMC3576830

[12] Cui Z Zhong S Xu P Gong G He Y. Panda: a pipeline toolbox for analyzing brain diffusion images. Front Hum Neurosci. (2013) 7:42. 10.3389/fnhum.2013.00042 PMC3578208 23439846

[13] Chen HJ Qi R Ke J Qiu J Xu Q Zhong Y, et al. White matter abnormalities in patients with typhoon-related posttraumatic stress disorder. Front Hum Neurosci. (2021) 15:665070. 10.3389/fnhum.2021.665070 PMC8511510 34658811

[14] Hua K Zhang J Wakana S Jiang H Li X Reich DS, et al. Tract probability maps in stereotaxic spaces: analyses of white matter anatomy and tract-specific quantification. Neuroimage. (2008) 39:336–47. 10.1016/j.neuroimage.2007.07.053 17931890 PMC2724595

[15] Smith SM Jenkinson M Johansen-Berg H Rueckert D Nichols TE Mackay CE, et al. Tract-based spatial statistics: voxelwise analysis of multi-subject diffusion data. Neuroimage. (2006) 31:1487–505. 10.1016/j.neuroimage.2006.02.024 16624579

[16] McCarthy P. Fsleyes. *Zenodo* (2022). 10.5281/zenodo.6511596

[17] Whitfield-Gabrieli S Nieto-Castanon A. Conn: a functional connectivity toolbox for correlated and anticorrelated brain networks. Brain Connect. (2012) 2:125–41. 10.1089/brain.2012.0073 22642651

[18] Murayama K Tomiyama H Tsuruta S Ohono A Kang M Hasuzawa S, et al. Aberrant resting-state cerebellar-cerebral functional connectivity in unmedicated patients with obsessive-compulsive disorder. Front Psychiatry. (2021) 12:659616. 10.3389/fpsyt.2021.659616 33967861 PMC8102723

[19] Jung J-Y Park C-A Lee Y-B Kang C-K. Investigation of functional connectivity differences between voluntary respirations via mouth and nose using resting state FMRI. Brain Sci. (2020) 10(10):704. 10.3390/brainsci10100704 PMC7599777 33022977

[20] Benjamini Y Hochberg Y. Controlling the false discovery rate: a practical and powerful approach to multiple testing. J R Stat Soc Ser B. (1995) 57:289–300. 10.2307/2346101

[21] Frizzell TO Phull E Khan M Song X Grajauskas LA Gawryluk J, et al. Imaging functional neuroplasticity in human white matter tracts. Brain Struct Funct. (2022) 227:381–92. 10.1007/s00429-021-02407-4 34812936 PMC8741691

[22] Mayes SD Calhoun SL. Learning, attention, writing, and processing speed in typical children and children with ADHD, autism, anxiety, depression, and oppositional-defiant disorder. Child Neuropsychol. (2007) 13(6):469–93. 10.1080/09297040601112773 17852125

[23] Low E Crewther SG Ong B Perre D Wijeratne T. Compromised motor dexterity confounds processing speed task outcomes in stroke patients. Front Neurol. (2017) 8:484. 10.3389/fneur.2017.00484 28983276 PMC5613174

[24] Heflin LH Laluz V Jang J Ketelle R Miller BL Kramer JH. Let’s inhibit our excitement: the relationships between stroop, behavioral disinhibition, and the frontal lobes. Neuropsychology. (2011) 25:655–65. 10.1037/a0023863 21574716 PMC3158285

[25] Bartzokis G Lu PH Tingus K Mendez MF Richard A Peters DG, et al. Lifespan trajectory of myelin integrity and maximum motor speed. Neurobiol Aging. (2010) 31:1554–62. 10.1016/j.neurobiolaging.2008.08.015 18926601 PMC2888859

[26] Torkamani-Azar M Kanik SD Aydin S Cetin M. Prediction of reaction time and vigilance variability from spatio-spectral features of resting-state eeg in a long sustained attention task. IEEE J Biomed Health Inform. (2020) 24:2550–8. 10.1109/jbhi.2020.2980056 32167917

[27] Tankus A Yeshurun Y Flash T Fried I. Encoding of speed and direction of movement in the human supplementary motor area. J Neurosurg. (2009) 110:1304–16. 10.3171/2008.10.Jns08466 19231930 PMC2837583

[28] Floden D Stuss DT. Inhibitory control is slowed in patients with right superior medial frontal damage. J Cogn Neurosci. (2006) 18:1843–9. 10.1162/jocn.2006.18.11.1843 17069475

[29] Dickinson D Ramsey ME Gold JM. Overlooking the obvious: a meta-analytic comparison of digit symbol coding tasks and other cognitive measures in schizophrenia. Arch Gen Psychiatry. (2007) 64:532–42. 10.1001/archpsyc.64.5.532 17485605

[30] Ho A Khan Y Fischberg G Mahato D. Clinical application of brain plasticity in neurosurgery. World Neurosurg. (2021) 146:31–9. 10.1016/j.wneu.2020.09.021 32916359

[31] Li L-M Zheng W-J Chen Y-Z Hu Z-H Liao W Lin Q-C, et al. Predictive factors of postoperative peritumoral brain edema after meningioma resection. Neurol India. (2021) 69:1682–7. 10.4103/0028-3886.333500 34979669

[32] Pascual-Leone A Amedi A Fregni F Merabet LB. The plastic human brain cortex. Annu Rev Neurosci. (2005) 28:377–401. 10.1146/annurev.neuro.27.070203.144216 16022601

[33] Duffau H Denvil D Capelle L. Long term reshaping of language, sensory, and motor maps after glioma resection: a new parameter to integrate in the surgical strategy. J Neurol Neurosurg Psychiatry. (2002) 72:511–6. 10.1136/jnnp.72.4.511 11909913 PMC1737830

[34] Leisman G Braun-Benjamin O Melillo R. Cognitive-motor interactions of the basal ganglia in development. Front Syst Neurosci. (2014) 8:16. 10.3389/fnsys.2014.00016 24592214 PMC3923298

[35] Middleton FA Strick PL. Basal ganglia output and cognition: evidence from anatomical, behavioral, and clinical studies. Brain Cogn. (2000) 42:183–200. 10.1006/brcg.1999.1099 10744919

[36] Frank MJ Loughry B O’Reilly RC. Interactions between frontal cortex and basal ganglia in working memory: a computational model. Cogn Affect Behav Neurosci. (2001) 1:137–60. 10.3758/CABN.1.2.137 12467110

[37] Grafman J. Conceptualizing functional neuroplasticity. J Commun Disord. (2000) 33:345–55; quiz 355–6. 10.1016/s0021-9924(00)00030-7 11001161

[38] Reed A Riley J Carraway R Carrasco A Perez C Jakkamsetti V, et al. Cortical map plasticity improves learning but is not necessary for improved performance. Neuron. (2011) 70:121–31. 10.1016/j.neuron.2011.02.038 21482361

[39] Morland AB Brown HDH Baseler HA. Cortical reorganization: reallocated responses without rewiring. Curr Biol. (2021) 31:R76–8. 10.1016/j.cub.2020.11.003 33497635

[40] Douglas DB Iv M Douglas PK Anderson A Vos SB Bammer R, et al. Diffusion tensor imaging of TBI: potentials and challenges. Top Magn Reson Imaging. (2015) 24:241–51. 10.1097/RMR.0000000000000062 26502306 PMC6082670

[41] Dennis EL Caeyenberghs K Hoskinson KR Merkley TL Suskauer SJ Asarnow RF, et al. White matter disruption in pediatric traumatic brain injury: results from enigma pediatric moderate to severe traumatic brain injury. Neurology. (2021) 97:e298–309. 10.1212/wnl.0000000000012222 34050006 PMC8302152

[42] Tu TW Williams RA Lescher JD Jikaria N Turtzo LC Frank JA. Radiological-pathological correlation of diffusion tensor and magnetization transfer imaging in a closed head traumatic brain injury model. Ann Neurol. (2016) 79:907–20. 10.1002/ana.24641 27230970 PMC4887193

[43] Winklewski PJ Sabisz A Naumczyk P Jodzio K Szurowska E Szarmach A. Understanding the physiopathology behind axial and radial diffusivity changes-what do we know? Front Neurol. (2018) 9:92. 10.3389/fneur.2018.00092 29535676 PMC5835085

[44] Bubb EJ Metzler-Baddeley C Aggleton JP. The cingulum bundle: anatomy, function, and dysfunction. Neurosci Biobehav Rev. (2018) 92:104–27. 10.1016/j.neubiorev.2018.05.008 29753752 PMC6090091

[45] Felderhoff-Mueser U Ikonomidou C. Mechanisms of neurodegeneration after paediatric brain injury. Curr Opin Neurol. (2000) 13:141–5. 10.1097/00019052-200004000-00005 10987570

